# From Lysosomal Storage to Neurodegeneration: Sphingolipid Signaling as a Driver of CNS Pathology and Biomarker Strategy in Neuronopathic Gaucher Disease

**DOI:** 10.3390/ijms27114788

**Published:** 2026-05-26

**Authors:** Krista Casazza, Reena V. Kartha, Jeanine R. Jarnes

**Affiliations:** 1 K2D2 Consulting, Bonita Springs, FL 34135, USA; 2 Advanced Therapies Department, University of Minnesota, Minneapolis, MN 55455, USA; rvkartha@umn.edu (R.V.K.); utzx0002@umn.edu (J.R.J.)

**Keywords:** Gaucher disease, sphingolipids, glucosylsphingosine (lyso-Gb1), neuroinflammation, biomarkers, neuronopathic

## Abstract

Gaucher disease is a prototypical lysosomal sphingolipid storage disorder caused by pathogenic variants in *GBA1*, resulting in glucocerebrosidase deficiency and accumulation of bioactive lipids, including glucosylceramide and glucosylsphingosine (lyso-Gb1). While non-neuronopathic Gaucher disease is effectively managed with enzyme replacement and substrate reduction therapies, neuronopathic forms remain largely refractory to treatment due to progressive central nervous system (CNS) involvement and limited penetration of current therapies across the blood–brain barrier. Disease pathobiology extends beyond lysosomal substrate accumulation to encompass dysregulated sphingolipid signaling, particularly sphingosine-1-phosphate (S1P)-mediated “inside-out” signaling, alongside neuroinflammation, oxidative stress, and glial activation, which collectively drive neurodegeneration. In this review, we synthesize current knowledge on sphingolipid metabolism and signaling in neuronopathic Gaucher disease and integrate these mechanisms into a three-tier, CNS-focused biomarker framework. The first tier comprises substrate-proximal markers of lysosomal burden (lyso-Gb1), which reflect GCase deficiency and correlate with systemic disease severity but incompletely capture CNS pathology. The second tier comprises markers of glial activation and neuroinflammation (glial fibrillary acidic protein [GFAP], glycoprotein non-metastatic melanoma protein B [GPNMB]), which reflect the downstream neuroimmune response to sphingolipid accumulation. The third tier comprises markers of neuroaxonal injury (neurofilament light chain [NfL]), which index irreversible neuronal damage as the terminal consequence of uncontrolled CNS disease. Together, these tiers map distinct but mechanistically interconnected stages of disease progression, from lysosomal dysfunction through glial activation to neuroaxonal loss, enabling stage-specific interpretation of biomarker signals that single-analyte approaches cannot provide. We further examine how S1P-mediated inside-out signaling links intracellular lipid dysregulation to extracellular neuroimmune and neurovascular responses and how the blood–brain barrier shapes compartment-dependent biomarker behavior across cerebrospinal fluid and blood. By grounding biomarker selection in this mechanistic cascade, the framework provides explicit criteria for pairing analytes across tiers, interpreting discordance between peripheral and CNS compartments, and designing multi-modal endpoints for clinical trials of CNS-penetrant therapies. Despite these advances, significant challenges remain, including limited longitudinal datasets, variability in assay methodologies, and incomplete validation of biomarkers as surrogates of CNS disease progression. Addressing these gaps will require harmonized, multi-modal approaches integrating biochemical, functional, and imaging measures. By positioning neuronopathic Gaucher disease as a model of sphingolipid-driven neurodegeneration, this review highlights opportunities for biomarker-guided therapeutic development relevant to Gaucher disease and the broader spectrum of sphingolipid-associated neurological disorders.

## 1. Introduction

### Sphingolipid Metabolism and Gaucher Disease Pathophysiology

Gaucher disease (GD; OMIM 230800) is a rare, inherited lysosomal disorder and is considered the most common lysosomal disease, with an estimated global prevalence of approximately 1 in 50,000–100,000 individuals and a U.S. birth incidence of roughly 1 in 40,000–100,000 [1]. GD is caused by pathogenic variants in the *GBA1* that reduce glucocerebrosidase (GCase) activity, resulting in impaired lysosomal hydrolysis of glucosylceramide (GlcCer) to ceramide and glucose and, consequently, accumulation of GlcCer and its deacylated metabolite glucosylsphingosine (lyso-Gb1) [2]. Substrate-laden macrophages (“Gaucher cells”) infiltrate multiple organs and drive systemic manifestations; diagnosis is established by demonstrating reduced GCase activity in leukocytes or fibroblasts and confirming *GBA1* variants by molecular testing [3]. Approximately 500 *GBA1* variants have been associated with GD, with p.Asn409Ser (p.N370S, (c.1226A>G) the most commonly observed variant among individuals with type 1 GD who are of European descent [4]. In contrast, the p.Asn409Ser mutation is rare in GD populations in India, China, Japan and Korea. The p.Asn409Ser mutation is also not common in Egypt, which has one of the largest GD populations in the world [4]. Clinically, GD phenotypes are often distinguished as non-neuronopathic (type 1) and neuronopathic or primary neuronopathic forms (types 2 and 3), the latter defined by progressive central nervous system (CNS) anomalies [4]. While enzyme replacement and substrate reduction therapies improve visceral and hematologic disease, CNS outcomes remain largely unchanged, consistent with limited CNS exposure due to blood–brain barrier constraints [4]. Primary neuronopathic GD (nGD) is heterogeneous in age of onset, symptom trajectory, and organ involvement, with geographic and genotypic variability reported across populations, including enrichment of severe genotypes such as p.L483P/p.L483P (p.L444P/p.L444P) in some regions [4].

This heterogeneity, together with limited longitudinal cohorts, has impeded the identification of predictors of neurological progression and the development of CNS-relevant drug development tools [4]. In addition, the ongoing expansion of newborn screening (NBS) programs for GD has highlighted that it may be difficult to distinguish between neuronopathic phenotypes (GD type 2 versus GD type 3), a determination that can be especially critical during the neonatal period if timely initiation of a life-saving therapy is required to ensure desired clinical outcomes [4]. Accordingly, advancing nGD therapeutics requires rigorously validated biomarkers that quantify CNS disease burden, track progression, and sensitively capture therapeutic response. Some candidate approaches include CSF and blood measures of lysosomal enzymes, sphingolipid metabolites (including lyso-Gb1), inflammatory mediators, and complementary neuroimaging and multi-omics strategies, ideally linked to standardized clinical outcomes. This review focuses on biomarkers proposed to reflect nGD pathophysiology and highlights key gaps limiting prognostic prediction. There is a particular focus on the inability to identify individuals at risk for rapid neurological decline and the need for higher-quality, systematic datasets to support clinical trial design and biomarker qualification [4,5].

Biomarkers are central to the diagnosis of GD, the monitoring of disease progression, and the evaluation of therapeutic efficacy; however, their development and validation remain particularly challenging in nGD. Although GD is fundamentally a disorder of sphingolipid metabolism, the contribution of downstream lipid signaling, inflammatory, and neurodegenerative pathways to disease activity and progression is incompletely understood [6]. Glucosylceramide (Gb1), the primary substrate of GCase, is a structural membrane lipid and a precursor of complex glycosphingolipids, while its deacylated, water-soluble derivative glucosylsphingosine (lyso-Gb1) accumulates through an alternative metabolic route and can diffuse beyond the lysosomal compartment [7]. Currently used biomarkers, including lyso-Gb1, reliably reflect systemic substrate burden but correlate inconsistently with clinical outcomes and neurological disease severity, particularly within the CNS [8]. Progress in biomarker development is further constrained by the rarity of nGD and the limited availability of well-characterized longitudinal cohorts, especially for GD types 2 and 3. Accordingly, advancing biomarker utility in nGD will require the identification of CNS-relevant markers, harmonization of analytical and clinical datasets, and integration of biomarkers with mechanistic and clinical evidence to support their use in therapeutic development and clinical trial design.

This review synthesizes current evidence regarding current and potential future candidates for fluidic, cellular, functional, and imaging biomarkers associated with neuronopathic GD, with a focus on their biological plausibility within sphingolipid metabolic and signaling pathways. Emphasis is placed on glucosylsphingosine as a pathogenic mediator and biomarker, as well as inflammatory and neurodegenerative markers linked to sphingolipid-driven immune activation. We further discuss challenges in biomarker validation, context-of-use qualification, and translational integration into clinical trials. By framing nGD as a model sphingolipid-mediated neurodegenerative disorder, this review highlights opportunities for biomarker-guided therapeutic development relevant not only to GD but also to a broader spectrum of sphingolipid-associated neurological diseases.

**Figure 1:**
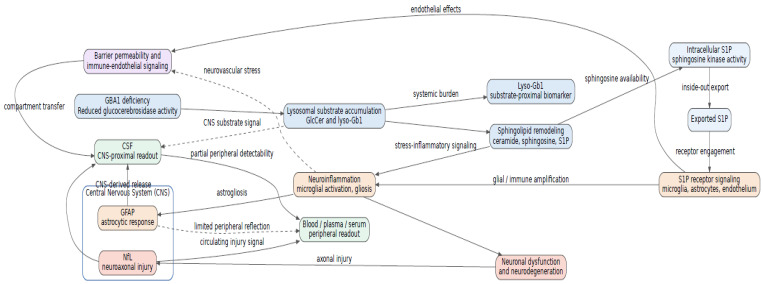
Integrated sphingolipid-driven pathobiology and three-tier biomarker framework in neuronopathic Gaucher disease. The schematic begins with a Gaucher macrophage/lysosomal compartment to emphasize substrate-driven initiation of the disease cascade. Reduced GCase activity results in lysosomal accumulation of glucosylceramide (GlcCer) and generation/accumulation of glucosylsphingosine (lyso-Gb1). These storage-associated perturbations are proposed to remodel downstream sphingolipid signaling pools (ceramide, sphingosine, sphingosine-1-phosphate [S1P]) and activate coupled stress–inflammatory programs. In the CNS, microglial activation and neuroinflammation amplify neuronal dysfunction, while “inside-out” S1P signaling (intracellular synthesis followed by export and receptor-mediated signaling through sphingosine-1-phosphate receptors (S1PRs), influencing microglial activation, endothelial barrier function, and neuroinflammatory amplification. The blood–brain barrier (BBB) modulates compartment-dependent biomarker transfer between the CNS, cerebrospinal fluid, and blood. Biomarker tiers are visually integrated into the mechanistic cascade: Tier 1 (blue) represents substrate accumulation and lysosomal dysfunction; Tier 2 (orange) represents glial activation and neuroinflammatory signaling; and Tier 3 (red) represents downstream neuroaxonal injury and neurodegeneration. Green compartments denote sampling domains (CSF and blood), while the purple interface represents the blood–brain barrier/neurovascular unit regulating compartment-specific biomarker transfer. Colors denote biological hierarchy: blue = substrate accumulation, orange = glial/inflammatory response, red = neuronal injury, green = peripheral sampling compartments, and purple = blood–brain barrier interface.

To facilitate interpretation of the heterogeneous biomarker landscape in neuronopathic Gaucher disease, this review adopts a three-tier mechanistic framework aligned with disease progression. Tier 1 biomarkers reflect substrate accumulation and lysosomal burden (e.g., lyso-Gb1); Tier 2 biomarkers reflect glial activation and neuroinflammatory signaling (e.g., GFAP, GPNMB); and Tier 3 biomarkers reflect downstream neuroaxonal injury and neurodegeneration (e.g., NfL). These tiers are integrated within the sphingolipid signaling cascade illustrated in Figure 1 and are discussed throughout the review in relation to compartment-specific behavior across CNS, CSF, and blood.

## 2. Search Criteria for This Review

- Trial registries searched: ClinicalTrials.gov and the EU Clinical Trials Register were queried to identify completed, terminated/withdrawn, and ongoing trials (recruiting/active not recruiting) involving nGD, yielding 19 nGD-relevant trials through December 2025.
- Databases searched (dates covered): PubMed/MEDLINE, Embase, and Scopus were searched from database inception through December 2025.
- Search strategy (keywords/MeSH): Searches combined controlled vocabulary and keywords for Gaucher disease and biomarkers, including terms for *GBA1*, sphingolipid metabolites (e.g., glucosylceramide, glucosylsphingosine/lyso-Gb1), lysosomal dysfunction, neurodegeneration, neuroinflammation, and neuroimaging (e.g., magnetic resonance imaging [MRI]; magnetic resonance spectroscopy [MRS]; diffusion tensor imaging [DTI]).
- Inclusion criteria: Studies in humans with suspected or confirmed nGD (types 2 or 3) evaluating candidate biomarkers measured in plasma/serum, dried blood spots, or CSF, and/or imaging biomarkers, with outcomes linked to neurological phenotype, disease severity, longitudinal change, and/or treatment response.
- Exclusion criteria: Studies limited to purely visceral type 1 GD without neurologic relevance (unless included as mechanistic or comparator cohorts) and nonclinical-only studies without clear translational relevance or linkage to candidate biomarkers evaluated in humans.
- Prioritization principle: We prioritized biomarkers supported by evidence linking analyte levels or imaging measures to neurologic phenotype, longitudinal progression, or therapeutic response.

Here, we review potential biomarkers identified through these criteria. Biomarker summary tables follow the terminology of the FDA-NIH Biomarker Working Group BEST (Biomarkers, EndpointS, and other Tools) Resource (https://www.ncbi.nlm.nih.gov/books/NBK326791/; accessed on 11 March 2026). “Proposed COU” (Context of Use) denotes the specific, prospectively defined role for which a biomarker is being evaluated; designations reflect current evidence level and do not imply regulatory qualification. Tables present five standardized domains: analytical matrix, primary biology reflected, proposed COU, key strengths, and key limitations.

## 3. Potential Fluidic, Cellular, Functional, and Imaging Biomarkers for nGD

nGD results from markedly reduced GCase activity, leading to lysosomal accumulation of glucosylceramide (GlcCer) and related sphingolipid metabolites that perturb lysosomal homeostasis and engage downstream inflammatory and neurodegenerative pathways. In the CNS, lipid accumulation within neurons and glia is associated with progressive neurological dysfunction, whereas systemic involvement reflects macrophage activation and Gaucher cell infiltration across organs. Consequently, biomarkers in nGD are needed to support (i) early diagnosis and phenotypic stratification, (ii) longitudinal tracking of neurological progression, and (iii) demonstration of CNS target engagement and therapeutic response, recognizing that circulating markers often reflect systemic substrate burden more reliably than CNS disease activity.

### 3.1. Considerations for Early Identification (Prenatal, Newborn, and Early Diagnostic Pathways) Biomarkers

Distinguishing neuronopathic from non-neuronopathic phenotypes remains a central challenge because genotype–phenotype correlations are incomplete and early clinical manifestations may be nonspecific [4,9]. Accordingly, screening paradigms have focused on enzymatic deficiency and substrate-proximal metabolites [9,10,11]. Prenatal evaluation in at-risk pregnancies typically measures GCase activity in amniotic fluid or chorionic villi; exploratory approaches have also proposed assessing macrophage activation markers, including chitotriosidase, in prenatal samples. In NBS, first-tier measurement of GCase activity in dried blood spots (DBS) by tandem mass spectrometry (MS/MS) or fluorescence-based methods provides a sensitive biochemical screen, with second-tier *GBA1* sequencing improving specificity and reducing false positives associated with pseudodeficiency alleles.

Clinical studies support this two-tier framework while underscoring limitations in predicting phenotype and therapeutic need. Kang et al. developed a fluorometric DBS method to detect *GBA1* activity and screened 80,855 newborns, identifying three infants with low activity using a 30.07 μmol·h^−1^·L^−1^ threshold; confirmatory leukocyte enzyme testing and *GBA1* analysis supported diagnosis and suggested feasibility for NBS implementation [12]. In a small neonatal series, Rasmussen et al. measured plasma Lyso-Gb1 (referred to as lyso-GL1) within 20 days of life and reported higher concentrations in neonates with nGD than in a neonate with non-neuronopathic GD; among NBS-identified cases, lyso-Gb1 values distinguished type 1 (0.016–0.12 nmol/mL; *n* = 4) from type 2 (0.883–1.792 nmol/mL; *n* = 2) and type 3 (0.417 nmol/mL; *n* = 1) and remained above the normal range despite treatment in their cohort [13]. Extending the utility of substrate-proximal measures to treatment decision-making at diagnosis, Dinur et al. [14] used DBS *GBA1* sequencing with lyso-Gb1 quantification at diagnosis in treatment-naïve pediatric patients and reported higher baseline lyso-Gb1 concentrations among those who subsequently initiated GD–specific therapy (60–1340 ng/mL) compared with those who did not (9–442 ng/mL). Receiver operating characteristic analysis supported a lyso-Gb1 cutoff >250 ng/mL (sensitivity 71%; specificity 87.5%) associated with treatment initiation, although inter-laboratory differences in assay methodology and reporting units limit generalizability of absolute thresholds [14]. Taken together, current screening and early diagnostic approaches are strongest for identifying GCase deficiency and systemic substrate burden, but they remain imperfect for distinguishing early-onset neuronopathic disease from milder phenotypes and predicting CNS trajectory. Enzymatic screening can be confounded by pseudodeficiency alleles and physiological variability, while second-tier genetic testing raises clinical and ethical challenges related to variants of uncertain significance and incidental risk disclosure (e.g., Parkinson disease predisposition) [15]. Beyond NBS, diagnosis after symptom onset is increasingly supported by mass spectrometry–based detection of disease-specific lipids (including lyso-Gb1) alongside confirmatory GCase activity and whole *GBA1* sequencing [4]. However, overlap in visceral and hematologic features with other lysosomal disorders (e.g., acid sphingomyelinase deficiency) reinforces the need for biomarker panels that improve diagnostic specificity and more directly capture CNS-relevant disease biology [16].

### 3.2. Sphingolipid-Derived and Sphingolipid-Associated Biomarkers

#### 3.2.1. Substrate-Proximal Biomarkers

**Glucosylsphingosine as a Bioactive Sphingolipid Mediator (Lyso-Gb1)**

**Mechanistic rationale.** Glucosylsphingosine (lyso-Gb1; also historically referred to as lyso-GL1) is the deacylated, more water-soluble derivative of glucosylceramide that accumulates in GD due to deficient GCase activity and altered lysosomal lipid handling [17]. Beyond its value as a substrate-proximal metabolite, lyso-Gb1 is plausibly positioned at the interface of lysosomal dysfunction and downstream sphingolipid signaling [4]. Following deglucosylation, lyso-Gb1 can contribute to sphingosine pools that are phosphorylated by sphingosine kinases to generate sphingosine-1-phosphate (S1P), a bioactive mediator implicated in immune cell trafficking and macrophage activation [18,19]. In parallel, experimental observations associate elevated lyso-Gb1 with cellular stress responses (e.g., ER stress, mitochondrial dysfunction, oxidative stress) and pro-inflammatory cytokine/chemokine induction, mechanisms consistent with amplified neuroinflammation. The mechanisms governing lyso-Gb1 synthesis and release from diseased tissue remain incompletely defined. One speculative mechanism proposed for lipid release into circulation is eructophagy, a process by which macrophages expel undigested lysosomal contents via autophagic machinery, originally characterized in the context of mycobacterial infection [20]. Whether analogous mechanisms operate in Gaucher macrophages to facilitate lyso-Gb1 release has not been directly demonstrated and warrants investigation.

**Clinical evidence.** Across multiple studies, lyso-Gb1 distinguishes GD from healthy controls and other lysosomal disorders and correlates with genotype and clinical severity in untreated disease. Dekker and colleagues reported elevated glucosylsphingosine in cerebrum and cerebellum tissue from individuals with nGD [21], consistent with earlier reports that glucosylsphingosine is typically absent from healthy brains yet detectable in GD-related neurological lesions, even when Gaucher cells are not observed in the CNS [22]. In a large Caucasian cohort, Rolfs et al. showed lyso-Gb1 discriminated Gaucher disease from controls using a threshold of 12 ng/mL and was higher in individuals with more severe genotypes (homozygous p.Leu483Pro, or p.L483P, also noted in the past as p.L444P) compared with p.Asn409Ser (or p.N409S, which has been noted as p.N370S in the past) [23]. Murugesan et al. observed markedly higher plasma lyso-Gb1 in untreated Gaucher disease (145.4–216.5 ng/mL) compared with controls (1.3–1.7 ng/mL) and demonstrated treatment-associated reductions with correlations to clinical indicators, including hepatosplenomegaly and inflammatory markers, with lower levels observed among eliglustat-treated patients in matched analyses [24]. In CNS-relevant datasets, Wilke et al. quantified lyso-Gb1 in both dried blood spots and CSF over cross-sectional and longitudinal follow-up and reported associations with age and other lysosomal/inflammatory measures, including CSF chitotriosidase [25].

**Limitations.** Lyso-Gb1 is a sensitive marker of systemic substrate burden and treatment engagement but should not be interpreted as a stand-alone surrogate of neurological severity or prognosis in nGD, given heterogeneity in phenotype, limited cohort sizes, and incomplete mechanistic validation linking circulating concentrations to CNS pathobiology (Table 1). Reported associations with genotype (e.g., p.Asn409Ser vs. homozygous p.Leu483Pro), inflammatory state, splenectomy status, and treatment modality introduce confounders that should be controlled in analyses and clinical trial stratification [23,24]. Methodological variability across laboratories, including differences in assay platforms (DBS vs. plasma vs. CSF; LC-MS/MS/UPLC-MS/MS) and units/reporting, limits the portability of absolute cutoffs and supports standardization efforts before lyso-Gb1 is used for cross-study qualification or regulatory-grade endpoint development. Finally, although CSF lyso-Gb1 provides a more proximate measure of CNS exposure than plasma, evidence remains insufficient to define how changes in CSF lyso-Gb1 map to clinically meaningful neurological outcomes in nGD, underscoring the need for harmonized longitudinal studies with standardized neurological endpoints [4].

#### 3.2.2. Neuroinflammatory and Glial Biomarkers

**Sphingolipid-Driven Neuroinflammation and Immune Activation**

Glycoprotein non-metastatic B (GPNMB).

*Mechanistic rationale.* GPNMB is upregulated in lysosomal stress states and appears to rise as part of a compensatory response to increased lysosomal burden. In nGD, GPNMB is expressed across relevant compartments, macrophages, microglia, and neurons, linking it to both systemic substrate-driven inflammation and CNS neuroimmune activation. In the CNS, elevated GPNMB is associated with microglial activation states that may reinforce a pro-inflammatory milieu and contribute to neurodegenerative features [24,26].

*Clinical evidence.* Using quantitative global proteomic profiling of CSF, Zigdon et al. [27]. reported that CSF GPNMB was significantly elevated in nGD and correlated with neurologic symptom severity. Consistent with a treatment-responsive profile, GPNMB has been observed to decrease following therapeutic intervention in available datasets; however, in the absence of large, well-controlled longitudinal nGD cohorts, this finding should be interpreted as supportive rather than definitive evidence of pharmacodynamic utility. GPNMB is elevated across other inflammatory contexts and has been proposed as a neurodegeneration-associated marker in other lysosomal and neurodegenerative diseases of the glycosphingolipid pathway (e.g., NPC, MPS VII models; Tay-Sachs/Sandhoff postmortem; ALS CSF), which complicates attribution to nGD biology alone (Table 2).

Chitotriosidase (CHITO)

*Mechanistic rationale.* CHITO, a human chitinase analog, is secreted by Gaucher cells and reflects macrophage activation and lysosomal dysfunction central to GD pathobiology. In nGD, macrophage-driven inflammation is relevant not only to visceral burden but also to neuroinflammatory tone, given systemic cytokine networks and immune–CNS crosstalk [28]. Oxidative stress, driven by glucosylceramide accumulation, misfolded GCase, and elevated ROS, contributes to lysosomal dysfunction and inflammation, processes that are closely associated with macrophage activation and elevated CHITO [29]. Gaucher macrophages release a broader inflammatory/lysosomal activation program in parallel (e.g., CCL18/PARC, interleukins, macrophage inflammatory proteins), situating CHITO within an interpretable “activated macrophage” biomarker axis.

*Clinical evidence*. Plasma CHITO activity is commonly used to monitor response to ERT. In a biomarker-modeling analysis, Vigan et al. incorporated clinical covariates (e.g., age at ERT initiation, splenectomy, genotype, sex, dose) and reported normalization of CHITO in ~65–66% of patients under modeled ERT effects, with substantial inter-patient variability and minimal covariate impact on CHITO change within that model [30]. These data support CHITO as a practical systemic response marker and help justify its continued inclusion in therapeutic monitoring panels (Table 3).

*Limitations.* The dominant limitation is interpretability for CNS disease. CHITO largely captures systemic macrophage burden and does not, by itself, localize pathology to neurologic compartments. Inter-patient variability remains substantial, and even when CHITO improves, residual inflammatory biology may persist (including oxidative stress–linked signals described in the broader biomarker narrative). Approximately 6% of the general population are homozygous for the *CHIT1* 24 bp duplication variant and exhibit virtually absent CHITO activity, while approximately 35% are heterozygous carriers with intermediate activity; both states can confound interpretation of CHITO in clinical monitoring [26,31,32].

### 3.3. CC Chemokine Ligand 18 (CCL18/PARC)

*Mechanistic rationale.* CCL18/PARC is secreted by Gaucher cells and rises with activation of lipid-laden macrophages, making it conceptually aligned with Gaucher cell burden and chronic immune activation. Plasma CCL18 is reported as ~10- to 50-fold higher in GD than in healthy subjects, consistent with persistent macrophage activation in response to accumulated glucosylceramide and lyso-Gb1 [33]. In nGD, CCL18 plausibly indexes an immune remodeling program relevant to tissue remodeling and neuroinflammation, given the role of macrophage/microglial activation in progressive neurological degeneration [31,32].

*Clinical evidence.* In adults with GD3 experiencing ERT dose reduction (ERT supply shortage due to viral contamination of the production plant during 2009–2010), Machaczka et al. [34] observed biomarker worsening. CCL18/PARC increased in 67% of patients (6/9) by +14% to +57% vs. baseline, while a subset remained stable or slightly decreased [34]. During the shortage window, patient-reported health status also shifted (EQ-5D domains), supporting clinical face validity that macrophage-associated biomarkers can move with treatment perturbations in GD3 (Table 4).

*Limitations.* CCL18/PARC is best positioned as a systemic macrophage-activation marker that reflects Gaucher cell burden and inflammatory tone; its relevance to CNS disease activity is therefore indirect and should not be interpreted as a CNS-specific biomarker in nGD [35]. In the GD3 enzyme-supply shortage analysis, CCL18/PARC increased in most patients, but not uniformly, and its trajectory was not fully concordant with CHITO across individuals, suggesting that macrophage-associated biomarkers can capture overlapping yet non-identical components of the inflammatory/lysosomal activation program. Additionally, CCL18/PARC elevation can occur as a general inflammatory marker in chronic inflammatory diseases and therefore is not exclusive to GD [31]. Accordingly, CCL18/PARC is most useful in multimodal panels (paired with CHITO and substrate-proximal markers such as lyso-Gb1) and interpreted alongside neurological endpoints rather than used as a stand-alone indicator of neurologic progression.

### 3.4. Neopterin

*Mechanistic rationale.* Neopterin is a pteridine produced by activated macrophages/monocytes in response to interferon-γ–linked immune activation and is often interpreted as a readout of Th1-type inflammatory tone. In nGD, glycolipid accumulation is associated with systemic and CNS inflammatory activation, including microglial engagement, making neopterin biologically plausible as an inflammation-linked marker [36,37]. Biochemically, neopterin production arises from GTP metabolism via GTP-cyclohydrolase I to a neopterin precursor, with secretion of neopterin and 7,8-dihydroneopterin from activated immune cells [38].

*Clinical evidence.* Drugan et al. reported increased plasma neopterin in untreated symptomatic GD1 patients versus controls and described normalization of mean neopterin with long-term ERT, while CHITO did not normalize [39]. They also found no correlation between CHITO and neopterin, suggesting these markers may diverge along the macrophage activation program and therefore provide complementary information rather than redundancy. Genotype-stratified analysis showed no significant differences across common genotypes, but neopterin trended higher in untreated patients carrying L444P or an unidentified mutation compared with N370S homozygotes [39] (Table 5).

*Limitations.* Neopterin is an immune activation marker with limited disease specificity. The primary utility of neopterin is contextual (inflammation burden and treatment-linked attenuation), not diagnostic discrimination. The CHITO–neopterin dissociation also underscores the need to avoid substituting neopterin for macrophage-burden markers; instead, it is better framed as a complementary inflammatory axis, ideally interpreted alongside clinical phenotype and other biomarkers.

### 3.5. α-Synuclein

*Mechanistic rationale*. α-Synuclein is relevant to Gaucher disease primarily through the broader lysosomal–proteostasis axis linking impaired glucocerebrosidase activity to altered autophagic protein clearance and increased Parkinson disease risk in GBA1-associated synucleinopathy. However, direct evidence supporting a pathogenic or biomarker role for α-synuclein in neuronopathic Gaucher disease remains limited.

*Clinical evidence*. Two studies provide the available human data, each with significant limitations for nGD inference that must be stated at the outset. Dubiela et al. studied α-synuclein mRNA in 65 ERT-treated GD patients, including a GD3 subgroup, and p.L444P/*GBA1* variant carriers and reported significantly elevated α-synuclein mRNA in GD3 patients and p.Leu483Pro (p.L483P) carriers compared with GD1 patients and controls, with GD1 patients showing mRNA levels similar to controls [40]. A positive age correlation was observed in p.L444P carriers but not in ERT-treated Gaucher patients. Critically, all GD patients in this cohort were receiving ERT at the time of sampling. ERT reduces lysosomal substrate burden and can modulate downstream proteostasis and stress-response pathways; accordingly, observed mRNA differences between phenotypic groups reflect a treatment-modified state rather than natural history and should not be interpreted as evidence that α-synuclein upregulation is an intrinsic feature of untreated nGD. Whether ERT attenuates, preserves, or exaggerates the α-synuclein signal relative to untreated disease is unknown. The genotype-linked signal in p.Leu483Pro (p.L483P) carriers, who were not all clinically affected, further complicates phenotype attribution, as carrier status and symptomatic nGD represent different biological contexts [40]. LoPiccolo et al. evaluated skin α-synuclein seeding activity (αSyn SAA) in adults with GD *type 1* and motor/cognitive phenotyping; αSyn SAA positivity was more prevalent than in historic controls, and the single GD1-PD case who underwent biopsy was positive [41]. This study provides proof-of-concept that tissue-based seeding assays are technically feasible in GD and that synucleinopathy biology is detectable at the periphery in some GD1 individuals. However, it offers no direct evidence regarding nGD: GD type 1 is by definition non-neuronopathic, the patients studied were adults with a disease course and PD-risk profile that differs fundamentally from GD types 2 and 3, and the neurodegenerative mechanisms driving GD2/3, acute brainstem neuronal loss and lysosomal neuronal death in infancy and early childhood are not the synucleinopathy-linked processes this assay is designed to detect. Extrapolation of αSyn SAA findings from GD1 adults to nGD should therefore be made explicitly as cross-phenotype hypothesis generation, not as evidence of a shared pathological mechanism [41] (Table 6).

*Limitations.* Current evidence supporting α-synuclein as a biomarker in nGD remains exploratory and indirect. Available studies involve heterogeneous populations, including treated GD1 cohorts and GBA1 variant carriers, limiting inference for untreated neuronopathic disease. Assay variability, uncertain CNS specificity, and the absence of longitudinal correlations with neurological outcomes further limit current clinical applicability.

### 3.6. Glial Fibrillary Acidic Protein (GFAP)

*Mechanistic rationale*. Astrocytic activation is increasingly recognized as a component of nGD neuropathology. Deficiency of GCase results in CNS accumulation of glucosylceramide and glucosylsphingosine, lipid species that promote lysosomal stress, neuroinflammatory signaling, and glial dysfunction [42]. GFAP represents a marker of astrocytic reactivity rather than a substrate-proximal biomarker.

*Evidence*. Experimental evidence supporting a relationship between nGD, astrocyte pathology, and GFAP derives from patient-derived cellular models. Induced pluripotent stem cell–derived astrocytes from individuals with nGD demonstrate abnormal sphingolipid accumulation, reactive astrogliosis, and increased GFAP expression, with more pronounced changes observed in acute neuronopathic disease. These findings suggest that astrocytes are not passive bystanders but active contributors to the neuropathological cascade in GCase deficiency, potentially amplifying neuronal injury through inflammatory and metabolic dysregulation [43]. However, direct clinical evidence linking GFAP to neurological disease burden remains limited (Table 7).

*Limitations*. While GFAP has emerged as a sensitive marker of astrocytic injury in other neurodegenerative and neuroinflammatory disorders, its performance varies by disease [44] and biological compartments [45], and peripheral GFAP concentrations do not uniformly reflect central nervous system pathology. CSF–based markers have shown closer associations with neurological involvement than blood-based measures, underscoring the importance of compartment-specific assessment.

Taken together, current data support a conceptual framework in which sphingolipid accumulation in nGD triggers astrocytic stress and reactive gliosis, reflected by increased GFAP expression at the tissue level. Future studies integrating sphingolipid profiling with astrocyte-specific biomarkers in paired CSF and blood samples are needed to determine whether GFAP provides incremental value for characterizing or monitoring neuronopathic involvement beyond established biochemical markers.

### 3.7. Neurofilament Light Chain (NfL)

*Mechanistic rationale.* NfL is a structural protein highly enriched in large-caliber myelinated axons and is released into cerebrospinal fluid and blood following neuroaxonal injury. As such, it functions as a non–disease-specific but highly sensitive marker of neuronal damage that can complement substrate-proximal sphingolipid measures that reflect lysosomal storage burden. In nGD, where central nervous system involvement is driven by GCase deficiency and sphingolipid accumulation, neurofilament light chain provides a conceptually distinct signal: downstream neuroaxonal injury rather than lysosomal substrate load.

*Clinical Evidence*. Recent prospective data in GD across subtypes showed that plasma neurofilament light chain was markedly elevated in type 2 disease and elevated in a subset of type 3 patients with clinically apparent neurological involvement, while type 1 patients had values within the reference range. In that study [46], combining neurofilament light chain with lyso-Gb1 and an abnormal auditory brainstem response improved early identification of severe neuronopathic phenotypes, including infants with minimal or absent neurological signs at presentation (Table 8).

*Limitation*. Because neurofilament light chain increases with age and is influenced by comorbid neurologic injury, its use in neuronopathic Gaucher disease should be framed as exploratory and interpreted in an age-adjusted manner, ideally with paired clinical anchors of neurological function and (when feasible) cerebrospinal fluid measurements.

### 3.8. Oculomotor

*Mechanistic rationale.* nGD preferentially involves brainstem, cerebellar, and basal ganglia circuitry that is critical for oculomotor control. Consistent with this neuroanatomical substrate, patients frequently exhibit impaired saccadic velocity and accuracy, gaze fixation instability, and smooth pursuit deficits that reflect disruption of brainstem burst neurons and cerebellar modulation, making eye-movement abnormalities a biologically plausible and CNS-proximal readout of neurological involvement [4]. Topo-anatomical patterns implicate the pontine paramedian reticular formation (PPRF), rostral interstitial nucleus of the medial longitudinal fasciculus (riMLF), abducens motoneurons, vestibular pathways, and cerebellar flocculus, supporting oculomotor testing as a functional marker of distributed CNS dysfunction in GD3 [47]. These measures are best viewed as functional endpoints rather than mechanistic biomarkers.

*Clinical evidence.* Bremova-Ertl et al. [48] performed cross-sectional and longitudinal video-oculography (EyeSeeCam^®^) in GD3 (*n* = 21) versus controls (*n* = 33), assessing reflexive saccades, smooth pursuit, gaze holding, optokinetic nystagmus, and vestibulo-ocular reflex (VOR) and relating performance to neurological scales (SARA, mSST) and a motor dexterity test (Grooved Pegboard Test). They reported compromised gaze-holding in a subset (consistent with cerebellar dysfunction) and significant correlations between clinical status and several eye-movement parameters, including peak velocity of downward saccades, vertical saccade duration, and horizontal angular VOR gains; over one year, the main longitudinal change was deterioration of vertical smooth pursuit [48]. Donald et al. similarly used EyeSeeCam to compare Type 1 GD (*n* = 39), Type 3 GD (*n* = 21), and controls (*n* = 35), confirming expected abnormalities in GD3 and unexpectedly detecting saccadic abnormalities in some GD1 individuals, reinforcing the phenotypic continuum relevant to trial inclusion criteria [49]. Benko et al. prospectively followed GD3 (*n* = 15; ages 8–28) for four years and demonstrated abnormal saccade main-sequence relationships (duration vs. amplitude; peak duration vs. amplitude) and increased horizontal saccadic latency, with downward saccades more affected than upward, and progression in a subset, consistent with slowly progressive neurological disease [50].

*Limitation*. Oculomotor measures provide sensitive, quantifiable functional assessment of CNS involvement and may be suitable as longitudinal endpoints, but they can vary across patients and may progress at different rates, necessitating standardized acquisition, rigorous training/quality control, and predefined analytic pipelines [4]. The primary value of oculomotor measures is as CNS functional readouts (progression/endpoint measures) rather than as direct indicators of specific molecular mechanisms, and they should be interpreted alongside clinical scales and complementary biomarkers (e.g., CSF/plasma analytes and imaging) to strengthen inference about disease biology and treatment effects.

### 3.9. Dysphagia

Dysphagia, i.e., impaired coordination of sucking/bolus preparation, swallowing, and breathing, reflects dysfunction of bulbar/brainstem and suprabulbar pathways and is a common, clinically consequential manifestation of GD2, with aspiration and feeding failure as major drivers of morbidity; it is also reported in GD3. Swallowing is classically conceptualized as oral, pharyngeal, and esophageal phases, and chronic impairment can lead to malnutrition, dehydration, and recurrent aspiration pneumonia. In GD2, Seehra et al. [51] developed a five-parameter swallowing evaluation that discriminated stages of neurologic decline, supporting swallow function as a pragmatic marker of disease progression and prognosis. Evidence from other neurodegenerative lysosomal disorders further supports swallow measures as clinically meaningful endpoints: in Niemann–Pick disease type C1, miglustat therapy was associated with improved/stabilized swallowing outcomes, a change with plausible downstream impact on aspiration-related complications, nutrition/hydration status, and quality of life [52].

### 3.10. Imaging Correlates of Sphingolipid-Induced Neurodegeneration

*Mechanistic rationale*. Neuroimaging and electrophysiologic measures provide noninvasive, CNS-proximal assessments of structural, microstructural, and functional changes associated with nGD, complementing fluid biomarkers that primarily reflect systemic burden. Because nGD involves heterogeneous CNS phenotypes with neurodevelopmental arrest and/or regression, MRI-based approaches (including volumetry, diffusion imaging, spectroscopy, and functional connectivity) are biologically plausible tools to quantify neurodegeneration and monitor progression. However, structural imaging alone may be insensitive in early disease and often differentiates neuronopathic from non-neuronopathic forms only after advanced atrophy emerges. Electroencephalography (EEG) provides a complementary functional readout of cortical and subcortical network integrity, capturing abnormalities in neuronal excitability and synchronization that are not detectable on structural imaging. In nGD, where seizures, brainstem dysfunction, and diffuse neuronal injury are common, EEG abnormalities, including background slowing, epileptiform discharges, and disorganized activity, may reflect early and dynamic CNS involvement [53]. As such, EEG is particularly well-suited to detect functional impairment preceding overt structural change and to monitor disease progression and treatment response in real time. Importantly, conventional MRI remains essential for identifying clinically actionable complications of nGD, including hydrocephalus, cranio-cervical junction stenosis, and compressive myelopathy, where timely detection can prevent irreversible injury [54]. Accordingly, an integrated framework combining MRI (structural and microstructural injury) with EEG (functional network disruption) is best positioned to capture the full spectrum of CNS involvement, linking neuroanatomical changes with real-time functional decline.

*Clinical evidence.* In a prospective pediatric study, Razek et al. evaluated children with GD2 (*n* = 7) and GD3 (*n* = 14) using proton magnetic resonance spectroscopy (^1^H-MRS) in frontal white matter and reported a significant difference in choline/creatine (Ch/Cr) ratios between nGD and controls, with a lipid peak detected in 15 nGD patients [55]. They also reported genotype- and phenotype-linked differences: acute nGD showed higher modified severity scoring tool (m-SST) and higher Ch/Cr than chronic forms and homozygous p.Leu483Pro (p.L483P) patients had higher m-SST and Ch/Cr than L444P/other; Ch/Cr was negatively correlated with m-SST [55]. Using diffusion tensor imaging (DTI) and tract-based spatial statistics, Davies et al. [56] identified microstructural white matter changes in nGD (*n* = 4) relative to controls, most prominently in the middle cerebellar peduncles. This anatomic correlate is plausibly aligned with ataxia, whereas GD1 patients (*n* = 3) showed diffuse nonspecific changes without a focal signature [56]. In a treatment-context exploratory analysis, Schiffmann et al. assessed volumetric MRI and resting-state fMRI during the venglustat plus imiglucerase trial and observed modest improvement in ataxia scores at Week 52, slight whole-brain volume increases in a subset of analyzable participants, and strengthened connectivity across distributed networks in most patients, alongside reductions in glucosylceramide with drug exposure; one participant demonstrated marked brain volume decline despite treatment [57].

*Limitations*. Current imaging evidence is constrained by small cohorts, heterogeneous protocols, and limited direct linkage between specific imaging changes and clinically meaningful neurological outcomes, which complicates qualification of imaging measures as surrogate endpoints. While advanced modalities (DTI, MRS, tensor-based morphometry, resting-state connectivity) show promise for detecting microstructural and network-level abnormalities, standardization of acquisition, analysis pipelines, and longitudinal sampling is essential, and imaging should be paired with harmonized clinical scales and fluid biomarkers to strengthen inference. Practically, MRI remains particularly valuable for monitoring actionable structural complications (e.g., hydrocephalus, cranio-cervical junction stenosis, cord compression) where imaging-guided intervention can mitigate permanent neurological damage [57,58].

## 4. Sphingolipid Signaling Convergence

Figure 1 provides a mechanistic overview linking these biomarker tiers across the disease cascade, illustrating how lysosomal substrate accumulation progresses through neuroimmune activation toward irreversible neuroaxonal injury. Sphingolipid-driven pathology in nGD is best understood as the convergence of lysosomal substrate accumulation with bioactive lipid signaling programs that regulate stress responses, innate immunity, and vascular barrier integrity. In addition to glucosylceramide and lyso-Gb1 accumulation, disease-relevant downstream remodeling of ceramide, sphingosine, and S1P pools may shift cellular programs toward inflammatory activation and impaired homeostasis [59,60,61]. This convergence is particularly relevant in the CNS, where glial activation and neurovascular unit dysfunction can amplify neuronal vulnerability.

A useful integrative framework is the ceramide/S1P signaling balance, in which ceramide-enriched states are commonly associated with stress signaling, apoptosis susceptibility, and altered autophagy, while S1P signaling promotes cell survival, immune trafficking, and endothelial barrier regulation in a context-dependent manner [61,62]. In GD, lyso-Gb1 has been proposed not only as a quantitative biomarker of substrate burden but also as a potential mediator capable of influencing immune and glial pathology, directly or indirectly through sphingosine/S1P-linked signaling pathways [61,63,64]. While definitive causal links in humans remain limited, preclinical and translational evidence supports the concept that sphingolipid remodeling can act as a feed-forward driver of neuroinflammation rather than a passive reflection of storage [61].

“Inside-out” S1P signaling links intracellular sphingolipid metabolism to communication between cells. Sphingosine-1-phosphate (S1P) is produced inside cells by sphingosine kinases and then exported through transporters such as SPNS2, creating extracellular gradients. These gradients activate S1P receptors (S1PRs) on nearby cells, including microglia, astrocytes, and endothelial cells in the brain. In the CNS, this signaling pathway helps regulate microglial activation, immune cell trafficking, and blood–brain barrier function—processes that are broadly implicated in neurodegeneration across multiple disease states [65,66,67]. Finally, sphingolipid signaling intersects directly with BBB physiology. S1PR signaling modulates junctional organization, cytoskeletal dynamics, and inflammatory responsiveness in endothelial cells, influencing permeability and immune cell entry under inflammatory conditions. In nGD, this perspective supports a coherent disease model in which lysosomal sphingolipid imbalance, immune activation, and neurovascular unit dysfunction form a coupled circuit that shapes both biomarker behavior (blood vs. CSF) and clinical phenotype [65,66,67]. This framework also motivates pathway-aligned biomarker prioritization (e.g., paired measurement of lyso-Gb1 with inflammatory and BBB-associated markers) and provides mechanistic justification for evaluating CNS-penetrant therapeutics that modulate sphingolipid signaling in addition to substrate burden [68,69].

## 5. Relevance to Other Sphingolipid Disorders and Neurodegeneration

nGD is a prototypical sphingolipid disorder, but the mechanistic architecture, i.e., lysosomal dysfunction coupled to inflammatory and neurovascular amplification, extends across multiple sphingolipidoses. Related lysosomal diseases (e.g., Niemann–Pick diseases and leukodystrophies) similarly exhibit substrate accumulation alongside secondary perturbations in ceramide/S1P signaling, glial activation programs, and autophagy–lysosome stress responses that contribute to neurodegeneration [70,71]. These shared nodes support the rationale for cross-disease biomarker concepts, particularly when biomarkers reflect convergent pathways (neuroinflammation, BBB integrity, oxidative stress) rather than a single stored lipid species [Figure 1]. The same convergence logic is increasingly relevant to common neurodegenerative conditions. Altered sphingolipid metabolism has been implicated in Parkinson disease, Alzheimer disease, and ALS, where lipid remodeling intersects with proteostasis impairment, neuroinflammation, and synaptic vulnerability [67,71]. In Parkinson disease specifically, GBA1-associated lysosomal dysfunction provides a well-established genetic bridge to broader neurodegeneration frameworks, reinforcing the clinical importance of sphingolipid-driven inflammatory and neurovascular mechanisms beyond Gaucher disease. This supports positioning nGD as a model system for biomarker-guided translation in sphingolipid-mediated neurodegeneration [72]. BBB biology further strengthens this cross-disorder relevance. Sphingolipid signaling, particularly S1P receptor pathways, regulates endothelial barrier stability and immune trafficking, which are shared amplifiers of CNS injury in both rare and common neurodegenerative diseases [66,73]. Accordingly, integrating BBB-relevant measures (e.g., CSF/plasma biomarker pairing, imaging correlates, and inflammatory markers) may improve phenotypic stratification and endpoint sensitivity in nGD trials and may also inform biomarker strategies in other sphingolipid-associated neurologic diseases.

S1P alterations may be compartment-specific in neurologic disease; for example, elevated CSF levels have been reported in early multiple sclerosis without corresponding increases in blood [44]. This observation underscores that peripheral sphingolipid measurements do not necessarily reflect central nervous system pathology. In nGD, there are currently no data demonstrating that blood S1P is a reliable biomarker of neuronopathic involvement, and available evidence does not support its use to infer central nervous system disease severity.

## 6. Therapies to Treat the CNS

nGD presents significant unmet medical and clinical needs due to its severe and progressive nature, limited therapeutic options, and the complexity of its management. Unlike the non-neuronopathic forms of Gaucher disease, which primarily involve visceral manifestations, nGD affects the CNS, leading to profound neurological symptoms such as oculomotor abnormalities, seizures, cognitive decline, spasticity, and developmental delays [74]. Although current ERT therapies fail to address the neurodegenerative aspects of nGD, due to limited ability for ERT to cross the BBB, the FDA expanded approval for imiglucerase ERT in January 2026 to include the treatment of non-neurological symptoms in children and adults with GD type 3, based on data showing significant improvement in hematologic, hepatosplenomegaly and pediatric growth parameters in response to imiglucerase ERT in patients diagnosed with GD type 3 [75]. The SRT, miglustat, can cross the blood–brain barrier but has not demonstrated the ability to significantly change the course of nGD and also has significant gastrointestinal side effects that can be dose-limiting for some patients [9]. Eliglustat SRT has demonstrated efficacy for GD type 1 and has regulatory approval for this indication as a first-line therapy [76,77]. However, eliglustat does not have bioavailability to the central nervous system, as it is prevented from entering the CNS by p-glycoprotein efflux at the BBB [9]. The investigational SRT, venglustat, crosses the blood–brain barrier and is well-tolerated by patients. A phase 2 open-label trial of venglustat in four Gaucher disease type 3 patients showed improvements in abnormal eye movements, tremors, seizures, ability to understand new or complex information, and ability to perform activities of daily living [57]. Results of a phase 2 trial of venglustat combined with imiglucerase ERT in patients diagnosed with GD type 3 showed a significant decrease in Lyso-Gb1 in the plasma and CSF after 1 year of treatment. Improvements were also observed in ataxia and MRI connectivity strength [57]. A phase 3 trial in patients diagnosed with GD type 3 compared lyso-Gb1in plasma and CSF, as well as Gb1 in plasma and CSF, in patients treated with either oral venglustat SRT, which crosses the BBB, versus imiglucerase intravenous ERT, which is understood to not appreciably cross the BBB [78]. Significant reductions in these biomarkers were observed in patients treated with venglustat versus those treated with imiglucerase. Biomarker results were supported by functional improvements in the motor skill assessment, modified Scales for Assessment and Rating of Ataxia (mSARA), and in a cognitive skill assessment tool, the Repeatable Battery for the Assessment of Neuropsychological Status (RBANS). These functional assessments provided important insight into the relationship of Gb1 and lyso-Gnb1 in plasma and CSF with cognitive skills (language, attention, visuospatial skills, immediate memory, delayed memory) and motor skills as measured by mSARA (gait, stance, fast alternating hand movement test, nose-finger test, finger chase test, heel-shin slide test, speech disturbance). Experimental approaches, such as small molecules designed to cross the BBB, gene therapies, and CNS-directed enzyme delivery systems, are under investigation. The pharmacological chaperone, ambroxol, has shown promise in preliminary studies for stabilizing GCase activity and potentially benefiting CNS pathology, but further validation is needed [79].

These data were considered compelling and led to FDA “Breakthrough Therapy Designation” for venglustat for GD type 3 in March 2026 (https://www.sanofi.com/en/media-room/press-releases/2026/2026-03-18-06-00-00-3257888; accessed on 28 April 2026). Although new brain-penetrant SRT and combination therapies for nGD are promising, there continues an urgent need for CNS-targeted therapies capable of modifying disease progression and mitigating neurological deficits [31]. Experimental approaches, such as small molecules designed to cross the BBB, gene therapies, and CNS-directed enzyme delivery systems, are under investigation. The pharmacological chaperone, ambroxol, has shown promise in preliminary studies for stabilizing GCase activity and potentially benefiting CNS pathology, but further validation is needed [79]. Moreover, early and accurate diagnosis remains a challenge due to the variability in clinical presentation and the lack of universally applied newborn screening protocols that include nGD-specific biomarkers [10,11,80].

Applications for real-world settings need to be incorporated into nGD biomarker selection. The routine feasibility of brain imaging and lumbar punctures to measure CSF biomarkers in infants and neurologically impaired patients is invasive, often requiring general anesthesia, thereby imposing further risks and diminished feasibility and limitations in access to care for patients. There is a need for outcome measures that can be readily employed during routine clinical evaluations and therapy monitoring. Bremova-Ertl et al. [48] showed correlation of severity of abnormal ocular movements with the SARA (Scales for Assessment and Rating of Ataxia) score [48]. Functional outcomes such as ocular evaluations, swallowing studies, and evaluation of ataxia and speech (e.g., Scales for Assessment and Rating of Ataxia, or SARA) provide potential ways to evaluate meaningful central nervous system function in real world settings [51,81]. Koto et al. (2023) surveyed caregivers for patients with GD1, GD2 and GD3 and reported significant caregiver burden for all GD phenotypes, with the highest burden in GD2 caregivers [82]. The Global Neuronopathic Gaucher Disease Registry (GUARDIAN) reports on a patient-led initiative to better identify meaningful clinical outcomes for nGD, with emphasis on quality of life and burden for patients and caregivers, as well as identification of meaningful functional clinical outcomes. Stakeholders include global GD experts from seven countries, additional clinician experts from around the world, representation from three pharmaceutical industries, and caregivers and patients. This ongoing project aims to improve understanding of nGD and improve collaboration of stakeholders, regulatory agencies and patients for more meaningful applications of real-world data to improve clinical research and clinical care outcomes.

## 7. Summary

nGD is not simply a disorder of lysosomal substrate accumulation but a disorder of sphingolipid signaling dysregulation in which lysosomal failure initiates a mechanistically coupled cascade of glial activation, neuroinflammation, and neuroaxonal injury that current therapies and biomarker strategies have not adequately addressed. This review has argued that understanding this cascade, and specifically the role of S1P-mediated inside-out signaling as a bridge between intracellular lipid imbalance and extracellular neuroimmune and neurovascular responses, is a prerequisite to developing biomarkers that reflect CNS disease biology rather than systemic macrophage burden alone. The three-tier framework proposed here is intended to provide a biologically ordered structure for interpreting biomarker behavior across disease stage and compartment, rather than viewing individual analytes in isolation.

The three-tier biomarker framework operationalizes that mechanistic understanding. Substrate-proximal markers, principally lyso-Gb1, quantify lysosomal burden and treatment engagement but are insufficient surrogates for neurological progression when used in isolation. Glial activation markers (e.g., GFAP and GPNMB) capture the neuroimmune response downstream of sphingolipid accumulation and provide a mechanistically distinct signal unavailable from lyso-Gb1 alone. Neuroaxonal injury markers (e.g., NfL) index the terminal consequence of uncontrolled CNS disease and are most sensitive to irreversible progression. Interpreted across tiers and paired with functional endpoints (e.g., oculomotor assessment, swallowing evaluation, ataxia scales such as SARA), this framework enables stage-specific characterization of disease activity that no single analyte or functional measure can provide independently. The ceramide/S1P signaling balance cuts across all three tiers: ceramide-enriched states associated with lysosomal stress feed forward into glial activation programs, while S1P receptor signaling at the neurovascular unit modulates the BBB permeability and immune trafficking that determine whether and how biomarker signals are detectable across CSF and blood compartments. Biomarker interpretation in nGD is therefore inherently compartment-dependent, and peripheral measurements of lyso-Gb1 or NfL should be understood as attenuated, BBB-filtered reflections of CNS processes rather than direct indices of CNS disease severity.

This framework has direct implications for clinical trial design. CNS-penetrant therapies, including investigational substrate reduction approaches such as venglustat, require endpoints that can detect target engagement at the CNS level, track downstream glial and neuroaxonal responses, and distinguish disease modification from symptomatic stabilization. The three-tier structure provides explicit criteria for endpoint selection and analyte pairing that prior biomarker reviews, which have cataloged candidate markers without a unifying mechanistic logic, have not offered. Realizing this potential requires resolution of the field’s most pressing methodological gaps: harmonization of assay platforms and reporting units across laboratories, expansion of longitudinal cohorts in GD types 2 and 3, and prospective validation of biomarker changes against standardized neurological outcome measures. Practical accessibility must also be preserved, particularly considering lumbar puncture and advanced neuroimaging impose burden and feasibility constraints in infants and neurologically impaired patients, reinforcing the clinical importance of blood-based biomarkers and functional assessments that can be obtained during routine evaluations.

Beyond GD, the mechanistic architecture, lysosomal sphingolipid dysregulation coupled to S1P signaling perturbation, glial activation, and neurovascular dysfunction are shared across sphingolipidoses and are increasingly implicated in common neurodegenerative conditions, including Parkinson disease, where *GBA1* variants provide a direct genetic bridge. nGD is therefore not only a disease requiring better biomarkers; it is a model system in which the causal chain from sphingolipid metabolic failure to neurodegeneration is unusually tractable and in which biomarker-guided therapeutic development can generate insights transferable to a broader spectrum of sphingolipid-associated neurological diseases. The psychosocial and financial burden borne by patients lends urgency to that translational agenda and underscores that the endpoint of biomarker development is not regulatory qualification alone, but earlier diagnosis, more accurate prognosis, and therapies that meaningfully alter the course of CNS disease.

## Figures and Tables

**Table 1:** Lyso Gb1.

**Matrix**	Plasma and dried blood spots (primary); CSF (CNS-relevant, emerging)
**Primary biology reflected**	Substrate accumulation due to GCase deficiency; lysosomal dysfunction; sphingolipid signaling (sphingosine/S1P axis); macrophage activation and systemic inflammation
**Proposed COU**	Diagnostic biomarker; systemic disease burden and treatment-response marker; exploratory CNS involvement marker in nGD
**Key strengths**	Highly sensitive and specific for Gaucher disease; robust discrimination from controls; correlates with genotype severity and systemic disease burden; responsive to ERT/SRT; measurable across multiple matrices
**Key limitations**	Not a validated surrogate of neurological severity; influenced by genotype, treatment status, and inflammatory state; inter-assay variability limits standardized cutoffs; limited evidence linking CSF levels to meaningful CNS clinical outcomes

**Table 2:** Glycoprotein non-metastatic B (GPNMB).

**Matrix**	CSF (primary); blood/plasma (supportive, if available)
**Primary biology reflected**	Lysosomal stress response; neuroimmune activation (microglia/macrophage-linked)
**Proposed COU**	CNS disease activity/severity association; exploratory treatment-response marker in nGD
**Key strengths**	Elevated in CSF in nGD and reported correlation with neurologic severity
**Key limitations**	Limited specificity; limited longitudinal nGD datasets; unclear relationship to clinical endpoints across cohorts

**Table 3:** Chitotriosidase (CHITO).

**Matrix**	Plasma/serum (activity); CSF (exploratory)
**Primary biology reflected**	Gaucher cell/macrophage activation; lysosomal burden (systemic)
**Proposed COU**	Systemic disease monitoring and pharmacodynamic response to ERT/SRT; supportive biomarker in nGD panels
**Key strengths**	Widely used clinically; responds to therapy; extensive historical comparability
**Key limitations**	Poor CNS specificity; high inter-individual variability; interpretation impacted by splenectomy, inflammation, genotype, and assay harmonization

**Table 4:** CC chemokine ligand 18 (CCL18/PARC).

**Matrix**	Plasma/serum
**Primary biology reflected**	Gaucher cell/macrophage activation; chronic inflammatory milieu
**Proposed COU**	Systemic disease activity and treatment-perturbation sensitivity; supportive biomarker in GD3/nGD monitoring
**Key strengths**	Markedly elevated versus controls; changes with treatment perturbation reported
**Key limitations**	Indirect CNS relevance; not fully concordant with CHITO across individuals; affected by other systemic inflammation and comorbidities

**Table 5:** Neopterin.

**Matrix**	Plasma/serum
**Primary biology reflected**	IFN-γ–linked monocyte/macrophage immune activation (Th1-type inflammatory tone)
**Proposed COU**	Complementary inflammation biomarker (adds orthogonal signal to CHITO/CCL18); supportive treatment-response marker
**Key strengths**	Elevated in untreated symptomatic patients; with GD type 1, mean normalization reported with long-term ERT; may provide nonredundant signal vs. CHITO
**Key limitations**	Low disease specificity; influenced by infections/autoimmune inflammation; unclear mapping to neurologic progression without longitudinal CNS-linked datasets

**Table 6:** α-Synuclein.

**Matrix**	Blood (mRNA); skin biopsy (seeding assays)
**Primary biology reflected**	Proteostasis/lysosomal dysfunction–linked synucleinopathy biology; PD-risk–associated pathway
**Proposed COU**	Exploratory stratification/risk marker (especially PD-related features); not endpoint-grade for nGD progression
**Key strengths**	Genotype/phenotype signal reported (elevated α-syn mRNA in GD3/L444P carriers); tissue seeding assays show feasibility
**Key limitations**	Limited relevance to core nGD monitoring; uncertain causal linkage in nGD; assay variability; outcomes depend on longitudinal validation

**Table 7:** GFAP.

**Matrix**	Cerebrospinal fluid (most direct for CNS compartment); blood (serum/plasma; ultrasensitive immunoassays); tissue/cell models (e.g., patient-derived astrocytes as supportive mechanistic evidence)
**Primary biology reflected**	Astrocyte activation/reactive astrogliosis and astrocytic injury (downstream glial response to CNS stress/inflammation rather than lysosomal substrate burden per se)
**Proposed COU**	Exploratory marker of CNS involvement/reactive gliosis in nGD; hypothesis-generating adjunct to substrate-proximal sphingolipids and clinical neurological anchors; not endpoint-grade without prospective, longitudinal validation
**Key strengths**	Strong mechanistic plausibility for astrocyte involvement in GCase deficiency; patient-derived Gaucher astrocyte models demonstrate astrogliosis with GFAP upregulation alongside substrate accumulation, supporting a Gaucher-relevant astrocytic signal
**Key limitations**	Limited direct clinical validation in nGD; compartment effects (CSF vs. blood) and uncertain peripheral contributions can complicate interpretation; substantial inter-assay/inter-platform variability and pre-analytical sensitivity limit cross-study comparability without standardization

**Table 8:** Neurofilament light chain (NfL).

**Matrix**	Blood (serum/plasma; ultra-sensitive immunoassays); CSF (if feasible); longitudinal sampling preferred
**Primary biology reflected**	Neuroaxonal injury/degeneration (downstream consequence of CNS pathology rather than lysosomal substrate accumulation per se)
**Proposed COU**	Exploratory marker for neuronopathic involvement and severity stratification; potential adjunct for early identification of severe neuronopathic phenotypes in newly diagnosed infants/children; not endpoint-grade without longitudinal validation
**Key strengths**	Measurable in blood; sensitive to neuroaxonal injury; prospective data show marked elevation in nGD; complements substrate-proximal sphingolipids (e.g., lyso-Gb1) by reflecting downstream neural injury
**Key limitations**	Non-specific; strong age dependence requiring age-adjusted interpretation; assay/platform variability and pre-analytical factors; unclear responsiveness to Gaucher-directed therapies and limited evidence for treatment-monitoring claims

## Data Availability

No new data were created or analyzed in this study. Data sharing is not applicable to this article.
